# Nitric Oxide Does Not Inhibit but Is Metabolized by the Cytochrome *bcc*-*aa*_3_ Supercomplex

**DOI:** 10.3390/ijms21228521

**Published:** 2020-11-12

**Authors:** Elena Forte, Alessandro Giuffrè, Li-shar Huang, Edward A. Berry, Vitaliy B. Borisov

**Affiliations:** 1 Department of Biochemical Sciences, Sapienza University of Rome, P.le A. Moro 5, 00185 Rome, Italy; 2 CNR Institute of Molecular Biology and Pathology, P.le A. Moro 5, 00185 Rome, Italy; 3 SUNY Upstate Medical University, 750 E. Adams St., Syracuse, NY 13210, USA; berryl@upstate.edu (L.-s.H.), berrye@upstate.edu (E.A.B.); 4 Belozersky Institute of Physico-Chemical Biology, Lomonosov Moscow State University, Leninskie Gory, 119991 Moscow, Russia

**Keywords:** enzyme, ligand binding, enzyme inhibition, nitric oxide, respiratory chain complexes, mycobacteria, *bcc-aa_3_* supercomplex

## Abstract

Nitric oxide (NO) is a well-known active site ligand and inhibitor of respiratory terminal oxidases. Here, we investigated the interaction of NO with a purified chimeric *bcc*-*aa*_3_ supercomplex composed of *Mycobacterium tuberculosis* cytochrome *bcc* and *Mycobacterium smegmatis* *aa*_3_-type terminal oxidase. Strikingly, we found that the enzyme in turnover with O_2_ and reductants is resistant to inhibition by the ligand, being able to metabolize NO at 25 °C with an apparent turnover number as high as ≈303 mol NO (mol enzyme)^−1^ min^−1^ at 30 µM NO. The rate of NO consumption proved to be proportional to that of O_2_ consumption, with 2.65 ± 0.19 molecules of NO being consumed per O_2_ molecule by the mycobacterial *bcc*-*aa*_3_. The enzyme was found to metabolize the ligand even under anaerobic reducing conditions with a turnover number of 2.8 ± 0.5 mol NO (mol enzyme)^−1^ min^−1^ at 25 °C and 8.4 µM NO. These results suggest a protective role of mycobacterial *bcc-aa*_3_ supercomplexes against NO stress.

## 1. Introduction

Nitric oxide (NO) is a gaseous free radical that, while exerting physiological functions at low concentrations, can have deleterious effects on the cell at high levels, being able itself or in combination with reactive oxygen species to damage proteins, lipids and nucleic acids. NO production by activated macrophages through the inducible form of the enzyme NO synthase (iNOS) is indeed a common host defence mechanism against infections. *Mycobacterium (M.) tuberculosis*, like several other microorganisms, has evolved the ability to sense this ligand and elicit defensive mechanisms [1], such as the downregulation of the host iNOS expression [2,3], the degradation of proteins damaged by NO and reactive nitrogen species [4] and the production of enzymes resisting and/or metabolizing the ligand. Two types of enzymes are mainly involved in bacterial NO detoxification, the NO-reductases (NORs) and the NO-dioxygenases (NODs). The former reductively metabolize NO to dinitrogen oxide (N_2_O) under anaerobic conditions, whereas the latter degrade NO to nitrate (NO_3_^−^) using O_2_ as co-substrate. Most NODs belong to the globin superfamily, such as flavohaemoglobins (flavoHb) and truncated haemoglobins (trHb) that detoxify NO with high efficiency in many microbes. Genes encoding flavoHb and trHb have been found in mycobacteria too. In *M. tuberculosis*, a truncated single domain haemoglobin (HbN) was found to effectively oxidise NO to harmless nitrate and protect the microorganism from nitrosative stress [5,6]. In *M. smegmatis*, a similar role is played by a flavoHb [7].

One of the main targets of NO is the respiratory chain that generates the proton motive force across the bacterial or mitochondrial membrane needed by ATP synthase for ATP production. Among the respiratory enzymes, terminal oxidases are the preferential targets of the ligand [8]. At nanomolar concentrations and in competition with O_2_, NO rapidly inhibits the activity of both prokaryotic and eukaryotic terminal oxidases, leading to energy deficiency and redox imbalance due to enhanced production of reactive oxygen species. The inhibition occurs rapidly and is reversible. Interestingly, if the ligand is removed from solution, the bacterial *bd* type-terminal oxidase recovers the activity faster compared to the studied haem copper oxidases, suggesting that *bd* oxidases can enhance bacterial tolerance to NO and related nitrosative stress [9].

As an obligate aerobe, *M. tuberculosis* possesses a flexible, branched electron transport chain sustaining O_2_ reduction [10,11]. Electrons pass through type II NADH dehydrogenase or other dehydrogenases to the menaquinone pool, and then to O_2_ through either a supercomplex formed by cytochrome *bcc* and *aa*_3_-type cytochrome *c* oxidase (*bcc*-*aa*_3_) or a *bd*-type terminal menaquinol oxidase [12,13,14]. Mycobacteria have no soluble cytochrome *c*, but the *bcc* complex displays a dihaem *c*-type cytochrome playing the role of the cytochromes *c* and *c*_1_ in canonical electron transfer chains. Therefore, formation of the *bcc*-*aa*_3_ supercomplex is necessary to mediate the direct electron transfer from menaquinol to O_2_ [15]. The *bcc*-*aa*_3_ is composed of the two different transmembrane complexes *bcc* and *aa*_3_. The *bcc* complex encoded by the *qcrCAB* operon transfers electrons from menaquinol to the *aa*_3_-type cytochrome *c* oxidase. It comprises cytochrome *b* (QcrB) containing two *b* haem groups, a Rieske-type high potential Fe_2_S_2_ iron-sulfur protein (QcrA), and a dihaem *c*-type cytochrome (QcrC). The *bcc* is a homologue of the mitochondrial cytochrome *bc*_1_ (Complex III) and the chloroplast *b_6_f* complexes. Like the *bc*_1_ and *b_6_f* complexes, the mycobacterial *bcc* complex is proposed to utilize the Q-cycle mechanism to build an electrochemical proton gradient. The second component of the *bcc*-*aa*_3_, the *aa_3_*-type cytochrome *c* oxidase, is encoded by the *ctaBCDE* operon. Of interest, the genes *ctaD* and *ctaC* (but not *ctaB* and *ctaE*) are in close proximity to the *qcrCAB* operon [13]. The oxidase carries four redox-active metal centers: Cu_A_, haem *a*, haem *a*_3_ and Cu_B_. The Cu_A_ center, composed of two copper atoms, is located on CtaC (subunit II or COX2) and is probably the immediate electron acceptor from *c*-type dihaem in QcrC. The low-spin haem *a* possibly accepts electrons from CuA and is located on CtaD (subunit I or COX1). Ultimately, the electrons are transferred to the binuclear center (also located on CtaD), composed of the magnetically coupled high-spin haem *a*_3_ and CuB ion, where O_2_ reduction takes place. CtaE (subunit III or COX3) seems to hold no redox cofactors. Intriguingly, the cryo-EM structure of the *bcc*-*aa*_3_ respiratory supercomplex from *M. smegmatis* has revealed the association of the copper superoxide dismutase (SOD) SodC, present as a dimer on the periplasmic side of the membrane, on the top of *bcc* dimer and in contact with the QcrA and/or QcrC cytochrome *cc* subunit [16,17]. Sod C was suggested to provide protection against superoxide, locally generated by the respiratory chain, similarly to mitochondrial SOD-2, which is found associated with the respiratory supercomplex CI–CIII_2_–CIV in *Caenorhabditis elegans* [18]. Like other haem-copper terminal oxidases [19,20,21,22,23,24,25,26,27], the mycobacterial *bcc*-*aa*_3_ supercomplex is proposed to act as a proton pump to generate the proton-motive force [16]. It is worth mentioning that there is a kinetic advantage associated with the formation of a supercomplex composed of complexes III and IV, as reported recently [28]. Importantly, genetic deletion by homologous recombination of *bcc*-*aa*_3_ is lethal for mycobacteria [29], pointing to a central role in energy metabolism of the *bcc*-*aa*_3_ branch of the electron transport chain.

Recently, a purification protocol was reported, yielding a stable and active chimeric supercomplex (*bcc*-*aa*_3_) consisting of the cytochrome *bcc* from *M. tuberculosis* and the *aa*_3_-type cytochrome *c* oxidase from *M. smegmatis* [30]. Here, we studied the interaction of such a purified supercomplex with NO. Surprisingly, we found that the enzyme not only resists inhibition by the ligand, but also rapidly metabolizes it. 

## 2. Results

### 2.1. Cytochrome bcc-aa_3_ is Resistant to NO Inhibition 

**Figure 1:**
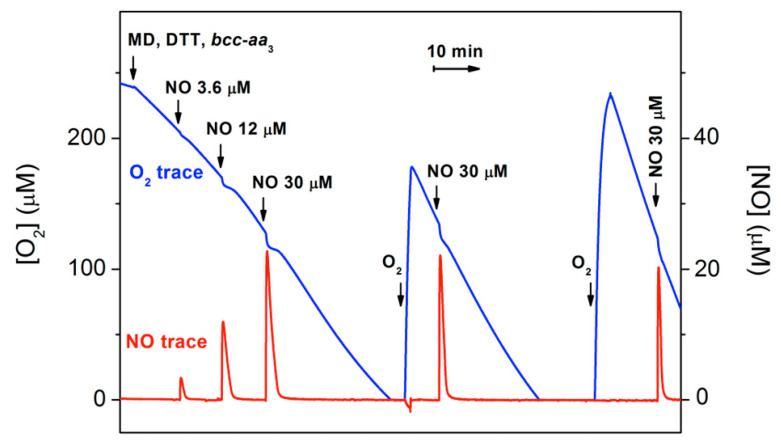
Effect of NO on O_2_ consumption by purified mycobacterial cytochrome *bcc-aa_3_* supercomplex. The addition of cytochrome *bcc-aa_3_* (67 nM) to the oxygraphic chamber containing the reducing system DTT/MD results in an O_2_ reductase activity of 144 e^−^ min^−1^. The effect of NO on the enzymatic O_2_ consumption is tested by sequentially adding to the chamber increasing volumes of NO-saturated water. Following O_2_ depletion and sample reoxygenation, O_2_ consumption re-starts and the activity increases up to 600 e^−^ min^−1^. Sequential additions to the 1.5-mL reaction chamber: 9.5 μL of 800 mM DTT (5 mM); 4 μL of 100 mM MD (0.26 mM); 10 μL of 10 μM cytochrome *bcc-aa_3_* (67 nM); 3, 10 and 25 μL of 1.8 mM NO (3.6, 12 and 30 μM, respectively).

The effect of NO on the dithiothreitol (DTT)/menadione (MD)-sustained O_2_-reductase activity of *bcc-aa_3_* was tested oxygraphically at 25 °C by simultaneously monitoring the concentration of O_2_ and NO in solution (Figure 1). Surprisingly, NO (even at relatively high concentrations) has little or no effect on the enzyme activity. As shown in Figure 1, only a small, transient decline in the O_2_ consumption is caused by NO additions, followed by quick and full restoration of the control O_2_-reductase activity of *bcc-aa_3_*. 

The activity recovery occurs as NO levels decline, the NO decay in the presence of the enzyme being faster than expected. Following O_2_ exhaustion and subsequent sample reoxygenation, the O_2_-reductase activity of *bcc-aa_3_* is enhanced (Figure 1). This phenomenon resembles the so-called ‘pulsing effect’ initially reported for the mammalian cytochrome *c* oxidase [31] and more recently documented for *Escherichia coli* cytochrome *bd*-I too [32]. It should be noted that after reoxygenation the enzyme retains its resistance to NO inhibition (Figure 1).

### 2.2. Cytochrome bcc-aa_3_ in Turnover Metabolizes NO 

**Figure 2:**
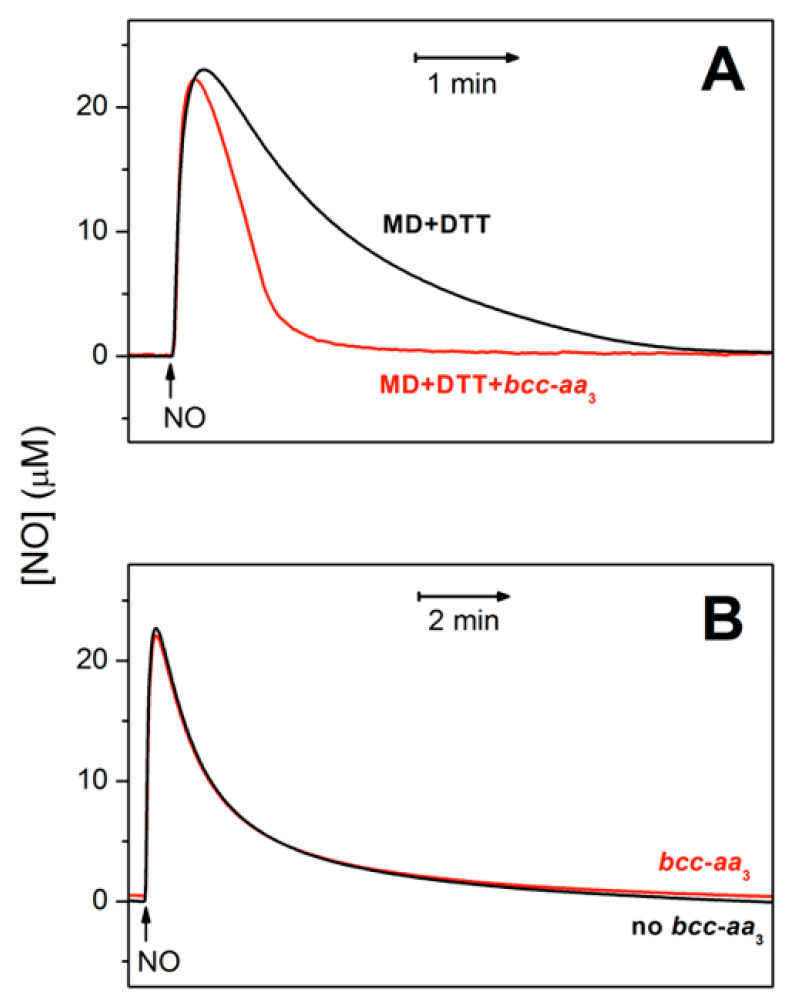
Purified mycobacterial cytochrome *bcc-aa*_3_ supercomplex in turnover conditions metabolizes NO. NO traces in the presence (red) and absence (black) of the supercomplex. (**A**) Cytochrome *bcc-aa*_3_ (67 nM) in turnover with the reducing system DTT (5 mM) and MD (0.26 mM) accelerates the decomposition of NO (30 µM) added to the chamber at [O_2_] ≈ 130 µM. At an initial NO concentration of 30 µM, the NO-consuming activity of the enzyme was ≈ 303 mol NO (mol *bcc-aa*_3_)^−1^ min^−1^. In the absence of the enzyme (black line) the NO degradation mainly occurs due to the reaction of NO with O_2_. (**B**) In the absence of DTT/MD, i.e., under non-turnover conditions, cytochrome *bcc-aa*_3_ (200 nM) does not accelerate the decomposition of added NO (30 µM).

Figure 2A shows that *bcc-aa_3_* in turnover with excess reductants and O_2_ can metabolize NO. In the presence of the supercomplex, the rate of NO decay is indeed significantly higher than measured in the absence of *bcc-aa_3_* under otherwise identical experimental conditions (Figure 2A, red trace vs. black trace). At an initial NO concentration of 30 µM, *bcc-aa_3_* displays a maximal NO-metabolizing activity of ≈303 mol NO (mol *bcc-aa_3_*)^−1^ min^−1^ at 25 °C, as estimated from initial rate analysis. The supercomplex can catalyze the aerobic degradation of NO only under turnover conditions. Indeed, as shown in Figure 2B, in the absence of reductants, the kinetics of NO decay with and without the enzyme in aerobic solution are virtually the same. 

**Figure 3:**
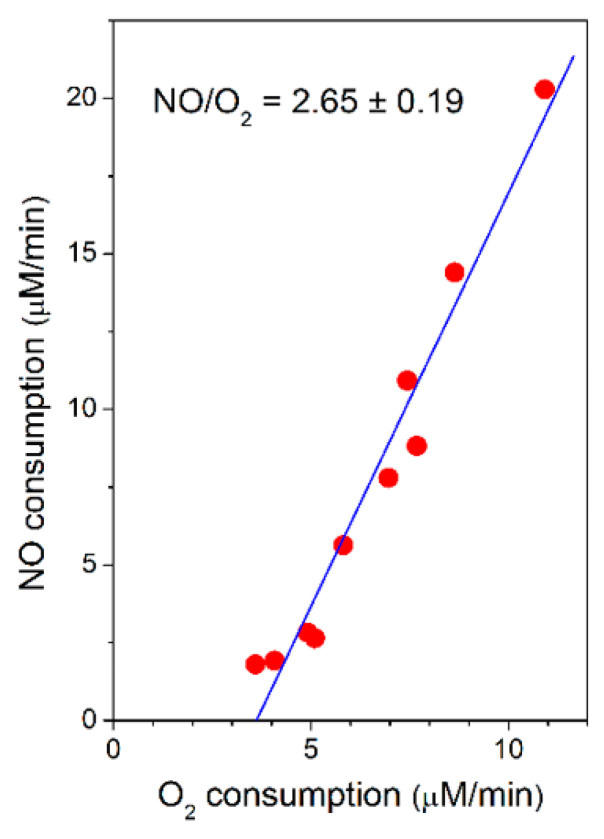
Rate of NO consumption plotted as a function of the rate of O_2_ consumption by purified mycobacterial cytochrome *bcc-aa_3_* supercomplex. NO (30 µM) was added to cytochrome *bcc-aa_3_* in the presence of DTT (5 mM) and MD (0.26 mM) at [O_2_] ≈ 130 µM. Conditions as in Figure 1. Linear regression analysis (solid line) of experimental data points (red circles: NO consumption rate) gives a NO/O_2_ ratio of 2.65 ± 0.19 (mean ± standard deviation).

Taking advantage of the spontaneous increase in the O_2_-reductase activity of *bcc-aa*_3_ observed along each oxygraphic assay (Figure 1), the rate of NO consumption by *bcc-aa_3_* could be measured at different actual rates of O_2_ consumption and the former was found to be proportional to the latter, according to a NO/O_2_ ratio of 2.65 ± 0.19 (Figure 3). 

### 2.3. Cytochrome bcc-aa_3_ Possesses NO-Reductase Activity 

**Figure 4:**
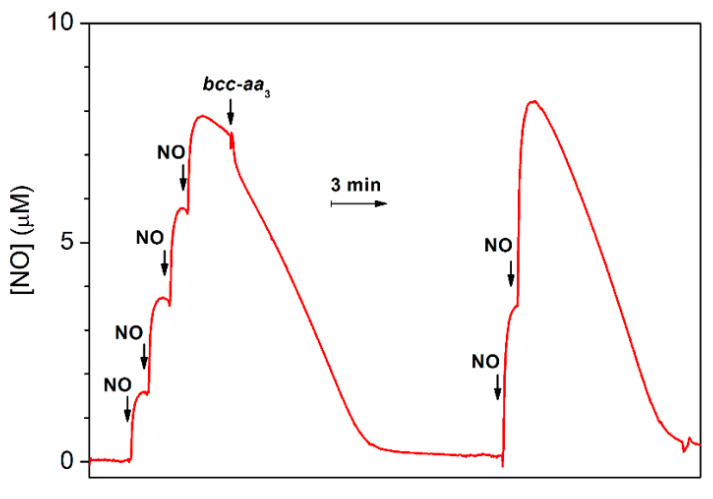
NO reductase activity of purified mycobacterial cytochrome *bcc-aa_3_* supercomplex. Four aliquots of 2.1 µM NO (1.8 μL of 1.8 mM NO each) were sequentially added to degassed buffer (buffer composition as in Figure 1) containing DTT (5 mM), MD (0.26 mM) as the reducing system, and glucose (5 mM) and glucose oxidase (16 units/mL) to scavenge residual O_2_ in the 1.5-mL reaction chamber. Then, 30 μL of 10 μM cytochrome *bcc-aa_3_* (200 nM), pre-reduced with DTT (5 mM) and MD (0.33 mM) in the presence of catalase (520 units/mL) and SOD (60 units/mL), was added to the chamber. The NO decay observed before addition of the supercomplex is likely due to the reaction with the reductants. Addition of pre-reduced cytochrome *bcc-aa_3_* accelerates the NO decay due to catalytic NO consumption. The initial fast drop in the NO concentration observed just after addition of the supercomplex is likely due at least partly to NO binding to cytochrome *bcc-aa_3_*. Following NO depletion from solution, two aliquots of 4.2 µM NO (3.5 μL of 1.8 mM NO each) were sequentially added and catalytic NO consumption further observed. The activity of *bcc-aa_3_* at [NO] = 8.4 µM was 2.8 ± 0.5 mol NO (mol *bcc-aa_3_*)^−1^ min^−1^. The calculated activity is expressed as mean ± standard deviation.

The ability of *bcc-aa*_3_ to degrade NO was also tested under anaerobic reducing conditions. In this assay, an aliquot of pre-reduced supercomplex was anaerobically added to an O_2_-free solution of NO containing excess reductants (DTT and MD), and the NO concentration in solution was then monitored using a NO-selective electrode. As shown in Figure 4, prior to enzyme addition, in the presence of excess DTT and MD, a slow decay of NO is observed due to reaction of NO with the reductants. The addition of *bcc-aa*_3_ clearly accelerates the decomposition of NO (Figure 4). The fast drop in the NO concentration observed immediately after addition of *bcc-aa*_3_ can be explained, at least partially, by NO binding to the reduced of *bcc-aa*_3_. The NO decay observed subsequently is consistent with a substantial catalytic NO reductase activity of *bcc-aa*_3_. After NO depletion, if the gas is re-added, the NO-consuming activity of *bcc-aa*_3_ is observed again (Figure 4). A notably slower NO consumption was observed if the same volume of aerobic buffer was added instead of the supercomplex in control experiments conducted under otherwise identical conditions (Supplementary Figure S1). The enzymatic NO reductase activity value was obtained from the slope of the trace with the enzyme after subtraction of the background non-enzymatic NO-reduction rate. Under anaerobic conditions, at [NO] = 8.4 µM, the estimated NO-reductase activity of *bcc-aa*_3_ proved to be 2.8 ± 0.5 mol NO (mol of *bcc-aa*_3_)^−1^ min^−1^ at 25 °C. 

## 3. Discussion

Clinical resistance of *M. tuberculosis* antibiotics represents an increasing threat to public health globally, preventing effective treatments against tuberculosis, one of the top 10 causes of death worldwide [33]. Interestingly, a correlation between resistance to first-line anti-TB drugs and reduced NO susceptibility has been found in clinical strains of *M*. *tuberculosis* [34]. Understanding how this bacterial pathogen resists the host immune system attack and identifying novel drug targets are therefore crucial for the successful cure of this infectious disease. The respiratory complexes of *M. tuberculosis*, including the *bcc-aa_3_* supercomplex, are attractive targets for the development of new antitubercular agents.

Kim et al. [30] recently succeeded in the purification of an untagged hybrid *bcc-aa_3_* supercomplex with *M. tuberculosis* cytochrome *bcc* and *M. smegmatis* cytochrome *aa*_3_. The supercomplex is stable and remains active during protein extraction and purification in the presence of the non-ionic detergent dodecyl-β-d-maltoside (DDM) [30]. This allowed us to use these *bcc-aa_3_* preparations for functional studies. Assays were meant to test whether *bcc-aa_3_* could contribute to mechanisms subverting, suppressing or evading the host immune response. In particular, we sought to gain insight into the reactivity of *bcc-aa_3_* with NO, a harmful species produced by the host as part of the immune response to fight microbial infections. To this purpose, we investigated the effect of NO on the O_2_ reductase activity of *bcc-aa_3_* and the ability of the supercomplex to metabolize NO under different conditions. 

First, we assayed whether the O_2_-reductase activity of *bcc-aa_3_* is affected by NO, which is known to bind with high affinity to the active site of respiratory terminal oxidases, resulting in a potent inhibition (reviewed in [35,36,37,38,39,40,41]). Surprisingly, we found that the mycobacterial supercomplex is resistant to NO inhibition, even at the highest concentration of the gas tested (30 µM). Such insensitivity to NO is unprecedented for a terminal oxidase. Indeed, the oxidases thus far assayed for NO inhibition, such as mitochondrial cytochrome *c* oxidase [42,43,44], the *aa*_3_ oxidase from *Rhodobacter sphaeroides* [45], the *cbb*_3_ oxidases from *Rhodobacter sphaeroides* and *Vibrio cholerae* [45] and the cytochromes *bd* from *Escherichia coli* and *Azotobacter vinelandii* [32,41,46], were found to be strongly and reversibly inhibited by NO with IC_50_ values in the nanomolar range. 

Figure 1 (blue trace) shows that the addition of 3.6 µM NO has no detectable effect on the O_2_ consumption by *bcc-aa_3_* in the presence of excess respiratory substrates. At the highest concentration of NO added, there is only a small decline in the O_2_ consumption followed by a relatively fast (≈1–2 min after the NO addition) and complete recovery of enzyme activity. The activity recovery occurs as NO disappears from solution (Figure 1, red trace). Careful comparison of the NO traces acquired in the presence and absence of *bcc-aa_3_* shows that the enzyme drastically accelerates the kinetics of NO decay (Figure 2A). The maximum NO-consuming activity of *bcc-aa_3_* detected at 30 µM NO and ≈130 µM O_2_ was ≈303 mol NO (mol *bcc-aa_3_*)**^−^**^1^ min^−^^1^. Thus, one can conclude that the apparent resistance of the supercomplex to NO inhibition results from its ability to directly metabolize NO, likely to nitrite (see below). NO consumption by the supercomplex requires turnover conditions, as *bcc-aa_3_* in the same aerobic buffer but without the DTT/MD reducing system proved to be unable to metabolize NO (Figure 2B). 

**Figure 5:**
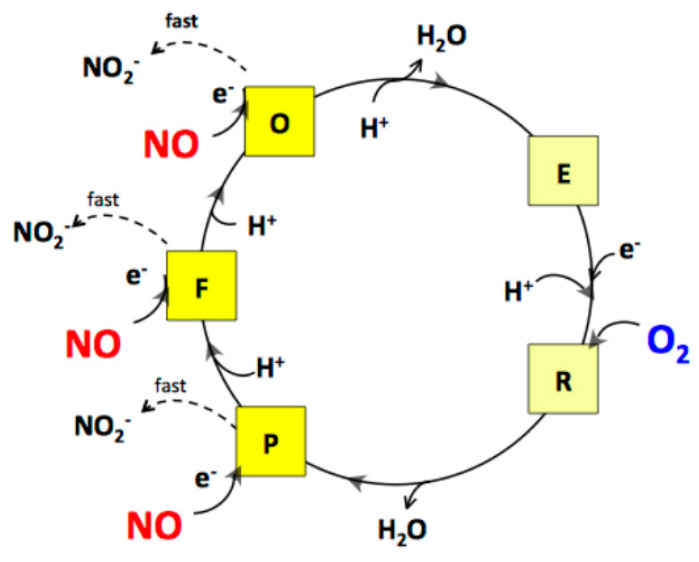
Proposed mechanism for the interaction of NO with the catalytic intermediates of the mycobacterial *bcc-aa_3_* supercomplex under aerobic conditions. As shown for mtCcOX, by reacting with the intermediates O, P and F, NO is suggested to lead to formation of nitrite. However, at variance from the mammalian enzyme, in the case of the mycobacterial supercomplex, the observed NO/O_2_ stoichiometry = 2.65 ± 0.19 suggests that nitrite does not bind with high affinity to oxidized haem *a_3_* and is rapidly ejected from the enzyme, without affecting its catalytic activity.

The rates of NO and O_2_ consumption catalysed by *bcc-aa_3_* are proportional, yielding a NO/O_2_ ratio of 2.65 ± 0.19 (Figure 3). In the bovine mitochondrial cytochrome *c* oxidase (mtCcOX), unlike O_2_ that binds only to the fully reduced (R) active site of the enzyme, NO was previously reported to bind and react also with the catalytic intermediates O (with the fully oxidized haem *a*_3_-Cu_B_ site), P (peroxy) and F (ferryl), each according to a 1:1 stoichiometry [47]. The reaction with the intermediates O, P and F is accompanied by injection of 1 electron into the enzyme from NO [47]. Donation of such an electron, while causing NO oxidation to nitrite, leads to conversion of an O_2_ intermediate into the succeeding one along the catalytic cycle (Figure 5). By reacting with NO, intermediates O, P and F are therefore respectively converted into the intermediates E (with the single electron-reduced haem *a*_3_-Cu_B_ site), F and O [47]. In mtCcOX, the nitrite formed from NO binds with relatively high affinity to the oxidized haem *a*_3_ in the active site, impairing the Fe reduction and, thus, its ability to react with O_2_. Consequently, the reaction with O_2_ stops. At lower reductive pressure on the enzyme, when intermediates O, P, F are more populated at steady-state, this inhibition pathway leading to the haem *a*_3_ Fe^3+^-NO_2_^−^ adduct prevails over the so-called nitrosyl pathway, that is instead favoured at higher reductive pressure and occurs via NO binding to reduced haem *a_3_*, leading to formation of a Fe^2+^-NO adduct (reviewed in [48]). Under the assumption that the mycobacterial *bcc-aa_3_* catalytic intermediates share a similar reactivity with NO, the observed >1 NO/O_2_ stoichiometry indicates that in the case of the mycobacterial enzyme NO can also react with more than one catalytic intermediate, but possibly with the notable difference (compared to the mitochondrial enzyme) that the newly formed nitrite does not bind to the haem *a*_3_ moiety with high affinity and, therefore, is ejected into the bulk phase from the enzyme without impairing its catalytic O_2_-reductase activity (Figure 5).

Interestingly, *bcc-aa_3_* was found to be capable of consuming NO also under anaerobic reducing conditions (Figure 4), with an estimated NO-reductase activity of 2.8 ± 0.5 mol NO (mol *bcc-aa_3_*)**^−^**^1^ min^−^^1^ (at [NO] = 8.4 µM and 25 °C), most likely leading to nitrous oxide (N_2_O) as the reaction product, similarly to other terminal oxidases. Indeed, the ability to catalyze the reduction of NO to N_2_O was previously documented under similar experimental conditions and setup (amperometric measurements under anaerobic reducing conditions at [NO] = 5–10 µM) in several haemcopper oxidases, namely in the *ba_3_* and *caa_3_* oxidases from *Thermus thermophilus* [49], the *cbb*_3_ oxidases from *Pseudomonas stutzeri* [50] and *Rhodobacter sphaeroides* [51], and the *bo*_3_ oxidase from *Escherichia coli* [52], but not in the mitochondrial beef heart cytochrome *c* oxidase [49,53] or the cytochromes *bd* from *Escherichia coli* and *Azotobacter vinelandii* [46]. Notably, the NO reductase activities of all terminal oxidases tested so far are significantly lower than that displayed by bacterial NORs. For instance, NOR purified from *Paracoccus denitrificans* shows the activity as high as ≈1400 mol NO (mol enzyme)**^−^**^1^ min^−^^1^ [50] (see also [54,55]). 

Taken together, the novel findings reported here, particularly the high resistance to NO inhibition of the mycobacterial *bcc-aa_3_* supercomplex and its unexpected NO-metabolizing activity, suggest a role for this enzymatic complex in the defence against NO, a major effector in the immune response to *M. tuberculosis* infection [56]. The resistance of *bcc-aa_3_* to NO inhibition may help the pathogen survive acute NO stress before transcriptional and late phase responses take place [57]. All in all, this study reveals unexpected properties of the mycobacterial *bcc-aa_3_* supercomplex, further pointing to this protein as a valuable drug target.

## 4. Materials and Methods 

### 4.1. Supercomplex Purification and Concentration Determination

The cytochrome *bcc-aa_3_* supercomplex was purified from a strain of *M. smegmatis* expressing the hybrid supercomplex, as described by Kim et al. [30] with modifications. Cells were collected by centrifugation. Membranes were prepared by sonication of cells in 50 mM K-Pi pH 7.4, 0.5 mM EDTA, and 0.1 mM phenylmethylsulfonyl fluoride (PMSF), followed by differential centrifugation, resuspended to 35 g/L protein in the same buffer, and frozen. For extraction, membranes were treated with DDM at a concentration of 10 g/L protein, 5 g/L detergent in 50 mM KPi pH 7.4, 100 mM NaCl, 0.1 mM PMSF. Insoluble material was separated from the extract by centrifugation. In this case the initial extract contained little soluble supercomplex but did have a large amount of soluble protein containing flavin and cytochrome *b_557_*. Therefore, the pellet was resuspended in buffer without detergent, protein content was measured, and the material was re-extracted as before but with 1 g/(g protein) of added DDM. This extract was diluted with ¼ volume of water, applied to DEAE-Sepharose CL6B column, and eluted with a gradient from 100 to 500 mM NaCl in 5-mM KPi, 0.5 mM EDTA, and 0.1 g/L DDM. Fractions containing *bcc-caa_3_* were identified by UV-visible spectra and haem-stained gels [58,59]. The pooled fractions were concentrated by ultrafiltration and layered on glycerol density step-gradients with 60, 40, and 20% *w*/*w* glycerol in 20 mM K-MPOS, 100 mM NaCl, and 0.5 mM EDTA, pH 7.2. The gradients were centrifuged at 30,000 rpm in the Beckman SW-32.Ti rotor for 72 h. Fractions containing *bcc-aa_3_* were pooled and chromatographed on Sepharose CL-6B size-exclusion medium equilibrated with 20 mM K MOPS, 100 mM NaCl, 0.5 mM EDTA, and 0.1 g/L DDM (pH 7.2). Fractions containing pure supercomplex, as assessed by SDS-PAGE (Supplementary Figure S2), were pooled and concentrated. Concentration of the supercomplex was estimated using the extinction coefficient for the oxidized form Δε_413-371 nm_ = 485 mM^−^^1^ cm^−^^1^. 

### 4.2. Catalytic Assays

Oxygraphic measurements were performed at 25 °C with a high-resolution respirometer (Oxygraph-2k, Oroboros Instruments) equipped with two 1.5-mL gas-tight thermostated chambers. The assays were carried out at 25 °C in 20 mM K/MOPS buffer (pH 7.3) containing 100 mM NaCl, 0.5 mM EDTA and 0.01% dodecyl-β-d-maltoside. Changes in the O_2_ and NO levels in solution were recorded in real time simultaneously. The NO concentration was recorded with the aid of a NO-selective electrode (World Precision Instruments, Sarasota FL, USA). The electrode was calibrated by sequential additions of NO from a stock solution, prepared by equilibrating degassed water at room temperature with pure NO (Air Liquide, Paris, France) at 1 atm. The O_2_ reductase activity of purified mycobacterial cytochrome *bcc-aa_3_* supercomplex was measured with an excess of the reductants DTT (5 mM) and MD (0.26 mM). Mycobacterial *bcc-aa_3_* preparations are indeed active with menadiol as substrate, which has a higher solubility in water solutions compared to menaquinol [60]. DTT was used to keep MD in the reduced state during the oxygraphic assays (Supplementary Figure S3). In the presence of DDT and MD, 520 units/mL catalase and 60 units/mL SOD were added to the solution to reduce the non-enzymatic background oxygen consumption.

### 4.3. Data Analysis

The catalytic NO-consuming activity was obtained by subtracting from the rate of NO consumption measured after addition of *bcc-aa_3_* from that of the non-enzymatic NO consumption. The latter was obtained by analyzing either the same trace prior to the enzyme addition (anaerobic reducing conditions with excess DTT and MD) or a trace independently collected under identical experimental conditions in the absence of *bcc-aa_3_* (aerobic conditions with excess DTT and MD). Origin software (OriginLab Corporation) was used for data analysis and figure preparation.

## Supplementary Materials

The following are available online at https://www.mdpi.com/1422-0067/21/22/8521/s1. Figure S1. Control NO trace acquired under anaerobic conditions in the absence of the supercomplex but in the presence of DTT/MD, Figure S2. SDS-PAGE analysis of the purified chimeric *bcc-aa_3_* supercomplex composed of *M. tuberculosis* cytochrome *bcc* and *M. smegmatis aa_3_*-type terminal oxidase Figure S3. Scheme illustrating the electron flow from DTT to O_2_ via MD and the cytochrome *bcc-aa_3_* supercomplex

## Abbreviations

NO	nitric oxide
TB	tuberculosis
iNOS	inducible NO synthase
NOD	NO-dioxygenase
NOR	NO reductase
flavoHb	flavohaemoglobin
trHb	truncated haemoglobin
SOD	superoxide dismutase
DTT	dithiothreitol
MD	menadione
mtCcOX	bovine mitochondrial cytochrome c oxidase
PMSF	phenylmethylsulfonyl fluoride
DDM	dodecyl β-d-maltoside
