# Supplementary material for: Nitric Oxide Does Not Inhibit but Is Metabolized by the Cytochrome bcc-aa3 Supercomplex
Source: Int J Mol Sci. 2020 Nov 12;21(22):8521. doi: 10.3390/ijms21228521 (PMC7697965; doi:10.3390/ijms21228521)
Supplement: Supplementary file 1 [file ijms-21-08521-s001.pdf]

International Journal of *Molecular Sciences*

MDPI

Supplementary materials for

Nitric oxide does not inhibit but is metabolized by the cytochrome *bcc-aa*3 supercomplex

Elena Forte, Alessandro Giuffrè, Li-shar Huang, Edward A. Berry, Vitaliy B. Borisov

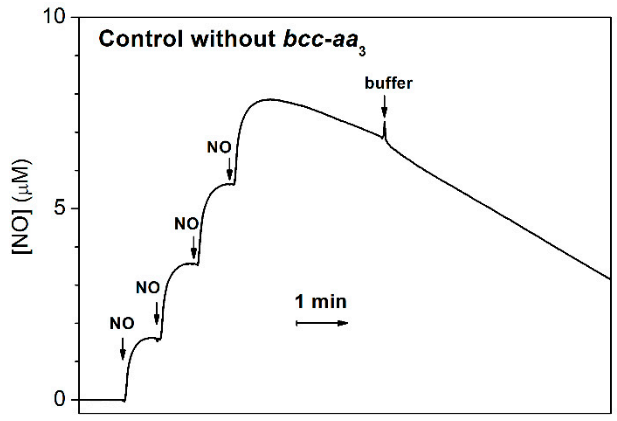

Supplementary Figure S1. Control NO trace acquired under anaerobic conditions in the absence of the supercomplex but in the presence of DTT/MD. Conditions as in Figure 4, except that an equivalent volume (30 μL) of air-equilibrated buffer (20 mM K/MOPS, 100 mM NaCl, 0.5 mM EDTA and 0.01% dodecyl-β-D-maltoside, pH 7.3) was added instead of the supercomplex.

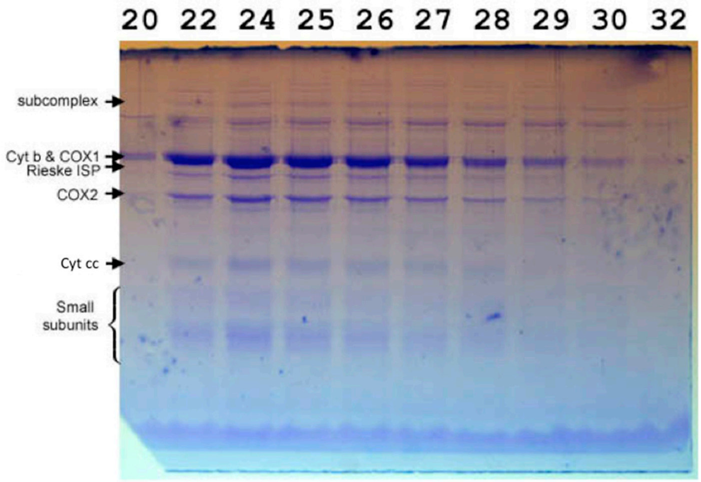

Supplementary Figure S2. SDS-PAGE analysis of the purified chimeric *bcc-aa*3 supercomplex composed of *M. tuberculosis* cytochrome *bcc* and *M. smegmatis aa*3-type terminal oxidase. Samples are the fractions from final gel-filtration column. Fractions 22-28 were pooled, concentrated and used for the assays. Under the mild denaturing conditions used, subunit dissociation is incomplete. Minor bands above cytochrome *b* (cyt *b*) and Cox I are probably subcomplexes, as is known to be the case for the band so labeled, which stains for heme indicating that it contains cytochrome *cc* (Cyt *cc*).

ISP: iron-sulfur proteins

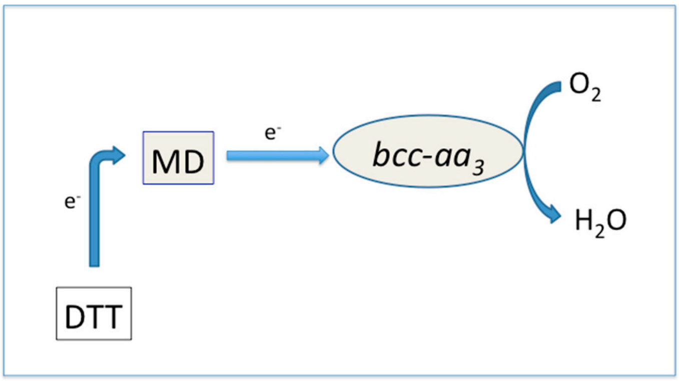

Supplementary Figure S3. Scheme illustrating the electron flow from DTT to O_2 via MD and the cytochrome *bcc-aa*$_3$ supercomplex. DTT reduces MD, which in the reduced state (menaquinol) binds at the quinol oxidation site of the supercomplex. In the supercomplex, the electrons donated by reduced MD are intramolecularly transferred to the heme a_3-Cu_B binuclear active site, where O_2 is reduced to H_2O.

Supplementary Method

Gel electrophoresis

Samples were denatured by mixing with an equal volume of the denaturing buffer described in [1] containing 2% SDS and 2% 2-mercaptoethanol and incubated at room temperature for several min before loading on a 80 × 60 × 0.75 mm 15% (T+C) polyacrylamide gel. The gel was run at 150 V in Laemli running buffer until the tracking dye reached the bottom, then stained first for heme (not shown) and then with Coomassie R-250 stain.

Reference

1. Laemli, U.K. Cleavage of Structural Proteins during the Assembly of the Head of Bacteriophage T4 ***Nature*** 1970, *227*, 680-685
